# A combination of cuproptosis and lncRNAs predicts the prognosis and tumor immune microenvironment in cervical cancer

**DOI:** 10.1007/s12672-024-00964-8

**Published:** 2024-04-12

**Authors:** Yitong Huang, Chenxiang Pan, Suni Wu, Feng Ye, Lihua Yang

**Affiliations:** 1 Department of Gynecological Oncology, Wenzhou Central Hospital, The Dingli Clinical Institute of Wenzhou Medical University, Wenzhou, 325000 Zhejiang China; 2 Department of Gynecology, Tangshan Maternal and Child Health Hospital, Tangshan, 063000 Hebei China

**Keywords:** LncRNAs, Cervical cancer, TME, Cuproptosis, Prognosis

## Abstract

**Background:**

Cuproptosis induces proteotoxic stress and eventually leads to cell death. However, the relationship between cuproptosis and lncRNAs in cervical cancer has not been fully elucidated. Therefore, we aim to explore the association among lncRNAs, cuproptosis and clinical features in cervical cancer.

**Methods:**

RNA sequencing, genetic mutations, and clinical data of CESC patients were obtained from TCGA. Cuproptosis-associated genes were gathered. WGCNA was used to cluster important modules, and KEGG, GO, GSEA and GSVA were used to explore functional and pathway enrichment. The association between immune microenvironment and cuproptosis-related lncRNAs was performed by using cibersort algorithm and other platforms, including XCELL, TIMER, QUANTISEQ, MCPCOUNTER and EPIC. Fluorescence quantitative PCR was employed to detect the expression of LINC01833 and LINC02321, and CCK-8 and cell scratch assays were used to assess cell proliferation and migration capabilities after LINCRNA interference.

**Results:**

202 upregulated and 45 downregulated lncRNAs were selected. The survival analysis showed that there was a statistically significant difference in survival rates between the high-risk and low-risk groups. The prognosis of tumour mutation burden and the degree of immune infiltration were differed noticeably between the high-risk and low-risk groups. BHG712, TL-2-105, FR-180204, Masitinib, TAK-715, ODI-027, JW-7-24-2, and OSI-930 had substantially higher IC50 values in the high-risk group. Notably, we found AL360178.1 was associated with RNF44 E3 ubiquitin ligase expression. In cervical cancer cell lines, LINC01833 and LINC02321 displayed significant upregulation. Efficient siRNA transfection led to a decreased expression of LINC01833 and LINC02321. This knockdown significantly hindered both cell proliferation and migration capabilities in cervical cancer cells compared to the negative control.

**Conclusion:**

In conclusion, we constructed five cuprotosis-related lncRNA prognostic models, which may be new tumor therapeutic targets for the prevention and treatment of cervical cancer.

## Introduction

Cervical cancer is one of the most common gynecological malignancies [1, 2]. The most common age of cervical carcinoma in situ is 30–35 years old, and the invasive cervical carcinoma is 45–55 years old [3, 4]. In recent years, the incidence of cervical carcinoma in situ is increasing in younger age [5, 6]. Most cervical cancer patients are associated with HPV infection [7]. Due to HPV vaccine and screening, the incidence of cervical cancer has decreased [8]. The prognosis of cervical cancer is closely related to clinical stage and pathological type [9]. The early stage of cervical adenocarcinoma is prone to lymphatic metastasis, so the prognosis of cervical cancer is worse than that of other gynecological tumors [10, 11]. Surgery, radiotherapy and chemotherapy are the main treatments for cervical cancer [5, 12]. But radiotherapy and chemotherapy can cause serious side-effects to patients and damage their own body functions [13, 14]. In addition, after repeated use of radiotherapy and chemotherapy, patients will develop drug resistance or tolerance, and the treatment effect will gradually decrease [15, 16]. Immunotherapy has been used in cervical cancer patients [17, 18]. There is an urgent need for an emerging novel treatment for cervical squamous cell carcinoma (CESC). Therefore, it is of great clinical significance to find reliable biomarkers to predict treatment response and prognosis, and to develop effective treatment strategies for CESC patients.

Depending on the mechanism of cell death, there are different ways of cell deaths, such as apoptosis, autophagy, necrosis, necroptosis, pyroptosis, cuproptosis, netosis, parthanatos and ferroptosis [19, 20]. Cuproptosis is a new type of cell death which was first discovered in March 2022 [21]. Copper is an indispensable trace element in all living organisms and is normally maintained at very low levels in mammalian cells [22]. Copper ions directly bind to the thioctylated component of the tricarboxylic acid (TCA) cycle, leading to abnormal aggregation of thioctylated proteins and decreased expression of Fe-S cluster proteins [23], which result in a proteotoxic stress response and cell death [24]. This novel approach of cell death may provide insight into the development of new therapies for CESC patients. Therefore, identifying the essential cuproptosis regulators is crucial for CESC therapy.

Noncoding RNAs (ncRNA) include ribosomal RNA, microRNA, transfer RNA (tRNA), circular RNA (circRNA), small interfering RNA (siRNA), piwi-interacting RNA (piRNA) and long noncoding RNA (lncRNA). Several reports have shown the critical role of miRNAs and circRNAs in cervical tumorigenesis as potential biomarkers and therapeutic targets [25, 26]. LncRNA is a noncoding RNA with a length greater than 200 nucleotides [27, 28]. LncRNAs can participate in cancer-related signaling pathways by regulating the expression of proto-tumor and tumor suppressor genes, and affect cell proliferation, tumor immune evasion, angiogenesis, apoptosis, tumor metastasis and a series of biological processes related to the cancer occurrence and development [29–32]. For example, lncRNA799 expression was considerably higher in cervical cancer tissue than in adjacent normal tissue, and its overexpression was associated with advanced stage, high SCC-Ag level, lymphatic metastasis, and poor survival [33]. In addition, recent studies have found that the changes of lncRNA expression and function may be related to apoptosis, autophagy, and ferroptosis [34–36]. One study showed that a lncRNA prognostic model associated with ferroptosis can be used to predict prognosis and provide immunotherapeutic targets for lung adenocarcinoma [37]. Tumor microenvironment (TME) comprises stromal cells, extracellular matrix, tumor cells and blood vessels, which is critically involved in cervical oncogenesis [38]. The role of lncRNAs in regulation of cuproptosis and TME has not been fully studied in cervical cancer cells. Therefore, the purpose of this study is to use bioinformatics analysis to reveal the relationship between cuproptosis, lncRNAs and clinical features in CESC.

## Materials and methods

### Data collection

We retrieved the transcriptome sequencing data and clinical information for 306 cervical cancer patients from the TCGA database. For further analysis, the 306 cervical cancer patients were randomly divided into the training (n = 153) and validation (n = 153) cohorts in a 1:1 ratio.

### Searching for cuproptosis-related lncRNAs

We collected 19 cuproptosis-related genes according to previous published papers. Using the "limma" package, we retrieved the list of cuproptosis-related genes and discovered the cuproptosis-related lncRNAs with |correlation coefficient|> 0.5 and a p-value < 0.05. After setting a p-value threshold of 0.05, we performed univariate Cox regression analysis to screen and detect prognostic cuproptosis-related lncRNAs. All analysis was carried out by R software (Version 4.1.3).

### Prognosis model of cuproptosis-related lncRNAs

The predictive significance of cuprotosis-related lncRNA characteristics was assessed using univariate and multivariate Cox regression analysis. The prognostic model accuracy was assessed using the receiver operating characteristic (ROC) curve and c -index. Following this, the data were separated randomly into two groups: the test group and the training group. The prognosis of the test group and the training group was then analyzed using a Kaplan–Meier curve. The prognostic lncRNAs related to cuprotosis were optimized using minimum absolute contraction and selection operator (LASSO) regression analysis, which also served to prevent data overfitting. We categorized the patients with cervical cancer into high risk and low risk groups based on the median risk score value of the training set. With the help of the "stats" and "Rtsne" R packages, PCA analysis was carried out.

### Construction of nomogram

Nomograms can be used to diagnose or predict disease onset or progression in combination with multiple indicators. To further improve the ability to predict long-term survival in CESC patients, a nomogram was constructed using the R package "rms" and a number of important clinical parameters and risk models.

### Functional enrichment of cuproptosis-related lncRNAs

The Kyoto Encyclopedia of Genes and Genomes (KEGG) is a comprehensive database integrating genomic, chemical and system functional information, aiming to reveal the genetic material and chemical blueprint of living phenomena [39, 40]. The Gene Ontology (GO) project is able to describe the biological functions of genes through a common semantic term. GO analysis mainly includes three levels: molecular function, biological process and cellular component. The package "clusterProfiler, ggplot2" was used to explore GO and KEGG functional enrichment analyses. GSEA (gene set enrichment analysis) is an unsupervised technique for assessing biological signatures at the gene set level. GSEA was performed on each sample in the cohort using the R packages "clusterProfiler", "enrichplot", and "DOSE". Gene set variation analysis (GSVA) was used to perform gene set (pathway) -level differential analysis by using the R package GSVA.

### WGCNA

Weighted correlation network analysis (WGCNA) is a systematic biological method used to describe gene association patterns between different samples. It can be used to identify highly synergistic gene sets and to identify candidate biomarker genes or therapeutic targets based on the interconnectedness of gene sets and the association between gene sets and phenotypes. Network construction, gene screening, gene cluster identification, topological feature calculation, data simulation and visualization are implemented by R package WGCNA.

### Infiltration of tumor-infiltrating immune cells

We used the CIBERSORT algorithm to construct a feature matrix from microarray data to describe the expression signatures of 22 immune cell phenotypes, including immune cells with different cell types and functional states. Gene expression signature set of 22 immune cell subtypes (LM22) was downloaded from https://cibersortx.stanford.edu/.

### Tumor microenvironment and immune cell correlation

The immune infiltration scores were calculated using the generally accepted methods, including XCELL, TIMER, QUANTISEQ, MCPCOUNT, EPIC, CIBERSORT, and CIBERSORT-ABS. The Wilcoxon signed-rank test was used to examine differences between two risk categories. We used the estimate algorithm to provide scores of tumor purity, stromal cell presence levels, and immune cell infiltration levels in tumor tissue. Furthermore, to acquire a better knowledge of the immunological milieu in different risk groups, we used the ggpubr R package to compare TME scores and immune checkpoint activation between risk categories.

### Analysis of tumor mutation burden

Tumor mutation burden (TMB) is defined as the total number of somatic gene coding errors, base substitutions, gene insertions, or deletions detected per million bases. TMB is thought to be a key driver in the production of immunogenic novel peptides that are expressed on cell membranes via major histocompatibility complexes and influence patient responses to immune checkpoint inhibitors. TMB was calculated using the "maftools" package. Furthermore, Pearson correlation analysis was used to calculate the association between the risk model and TMB.

### HPA

The HPA database (https://www.proteinatlas.org/) provided us with an immunohistochemistry expression graph of linked genes. Multiple genes are differently expressed in cancer, and many of them influence patient survival.

### Drug sensitivity analysis

Predicting drug susceptibility helps to aid in the selection of potential targeted therapy drugs based on genetic analysis, thereby improving the efficiency and precision of treatment. We used the pRRophetic package to predict phenotypes from gene expression data (clinical outcomes using Cancer Genome Project (CGP) cell line data) and to predict drug sensitivity in external cell lines (CCLE). The correlation between risk score and drug sensitivity was calculated by the R package WGCNA according to the expression level and IC50.

### Cell culture and transfection

The siRNAs were purchased from GimaGene (China), and their specific sequences are as follows. For LINC01833-siRNA-S, the sequence is GGCAUGGUCAGAGAAAGAATT, and for LINC01833-siRNA-AS, the sequence is UUCUUUCUCUGACCAUGCCTT. For LINC02321-siRNA-S, the sequence is GUUCCUUUCAACCAGCCAATT, and for LINC02321-siRNA-AS, the sequence is UUGGCUGGUUGAAAGGAACTT. Transfection was carried out using Lipofectamine 2000 (Thermo, United States) following the manufacturer’s protocol. For a 12-well plate, cells at a growth density of 80–90% were ideal for transfection. The day before transfection, cells were plated at a density of 2 × 10^5^ cells per well.

### Real-time PCR validation of transfection efficiency

Following PCR amplification, the real-time fluorescence quantitative PCR instrument automatically analyzed the results. Threshold and baseline adjustments were made based on negative controls to determine the Ct values for each sample. The validity of each Ct value was confirmed using the melt curve analysis. The results were exported, and the 2^−ΔΔCT^ method was employed to analyze the differential expression of the target gene between the control group and various experimental groups.

### Cell counting kit-8 (CCK-8) assay

Cell proliferation ability was assessed using the CCK-8 assay. Hela cells were seeded at a density of 9000 cells per well in a 96-well plate and incubated in a cell culture incubator for 24 h. Subsequently, siRNA for LINC01833 and LINC02321 were separately transfected, and CCK-8 assays were conducted at 24, 48, and 72 h post-transfection [41].

### Cell scratch assay

HeLa cells were seeded at a density of 1,200,000 cells per well in a 6-well plate and incubated in a cell culture incubator for 24 h. After 24 h, siRNA for LINC01833 and LINC02321 were separately transfected, and a scratch was made during medium changes. Photographs were taken at 0 h, 12 h, and 24 h, and experimental data were collected and analyzed [42].

## Results

### Differential expression of cuproptosis-related lncRNAs

**Fig. 1:**
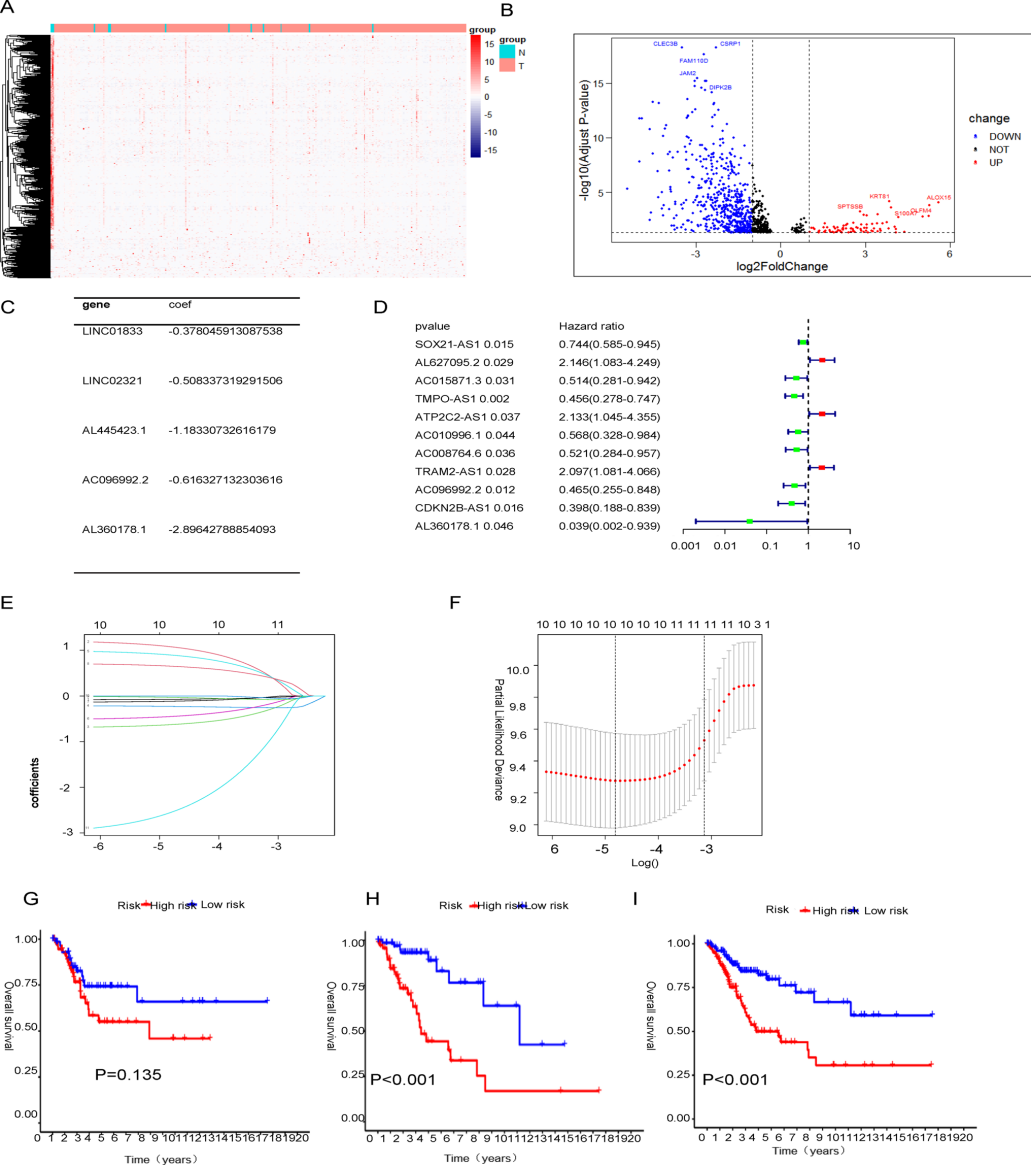
Differential expression of cuproptosis-related lncRNAs. **A** Heatmap of the differentially expressed genes in cervical cancer. The color scale indicates the level of gene expression, with red indicating upregulation and blue indicating downregulation. **B** Volcano plot showing the differentially expressed genes in cervical cancer. The red dots represent upregulated genes, the blue dots represent downregulated genes, and the black dots represent genes with no significant difference. **C** Univariate Cox regression analysis of clinical factors associated with patient survival in cervical cancer. The hazard ratio (HR) and 95% confidence interval (CI) are shown for each variable. **D** Multivariate Cox regression analysis of clinical factors associated with patient survival in cervical cancer. The HR and 95% CI are shown for each variable. **E** LASSO coefficient profiles of the cuproptosis-related lncRNAs. The dotted vertical line represents the optimal value of lambda, which was determined using tenfold cross-validation. **F** LASSO cross-validation plot showing the relationship between the log(lambda) and the mean squared error (MSE) of the model. **G** Kaplan–Meier curves of overall survival in the test group. The red and blue curves represent high- and low-risk patients, respectively. **H** Kaplan–Meier curves of overall survival in the train group. The red and blue curves represent high- and low-risk patients, respectively. **I** Kaplan–Meier curves of overall survival in the combined group. The red and blue curves represent high- and low-risk patients, respectively

This study included 306 CESC samples. The heatmap of differential expression of CESC-related lncRNAs was described (Fig. 1A). By setting the FDR < 0.05 as the cut-off criterion, 202 upregulated and 45 downregulated lncRNAs were shown in a volcano plot (Fig. 1B). We also conducted a co-expression analysis between cuproptosis and lncRNAs. With p-value less than 0.001 as the standard, we screened 11 lncRNAs associated with cuproptosis by cox regression analysis, which can predict the prognosis of cervical cancer. Subsequently, we used multivariate cox regression to construct a cuproptosis-related lncRNA model to predict the prognosis of cervical cancer. The model is as follows: risk score = (− 0.378045913087538 × LINC01833) + (− 0.508337319291506 × LINC02321) + (-1.18330732616179 × AL445423.1) + (− 0.616327132303616 × AC096992.2) + (− 2.89642788854093 × AL360178.1) (Fig. 1C, D). At the same time, we did lasso regression analysis to prevent overfitting (Fig. 1E, F). The samples were randomly divided into 1:1 training group and test group according to the median risk score. In the test group, there was no restricted difference between the low-risk group and the high-risk group, and the p-value was greater than 0.05 (Fig. 1G). The results of K-M survival analysis in the training group showed that the prognosis of the low-risk group was better than that of the high-risk group (Fig. 1H). Similarly, the outcomes of the low-risk group were differed from those of the high-risk group across the dataset (Fig. 1I).

**Fig. 2:**
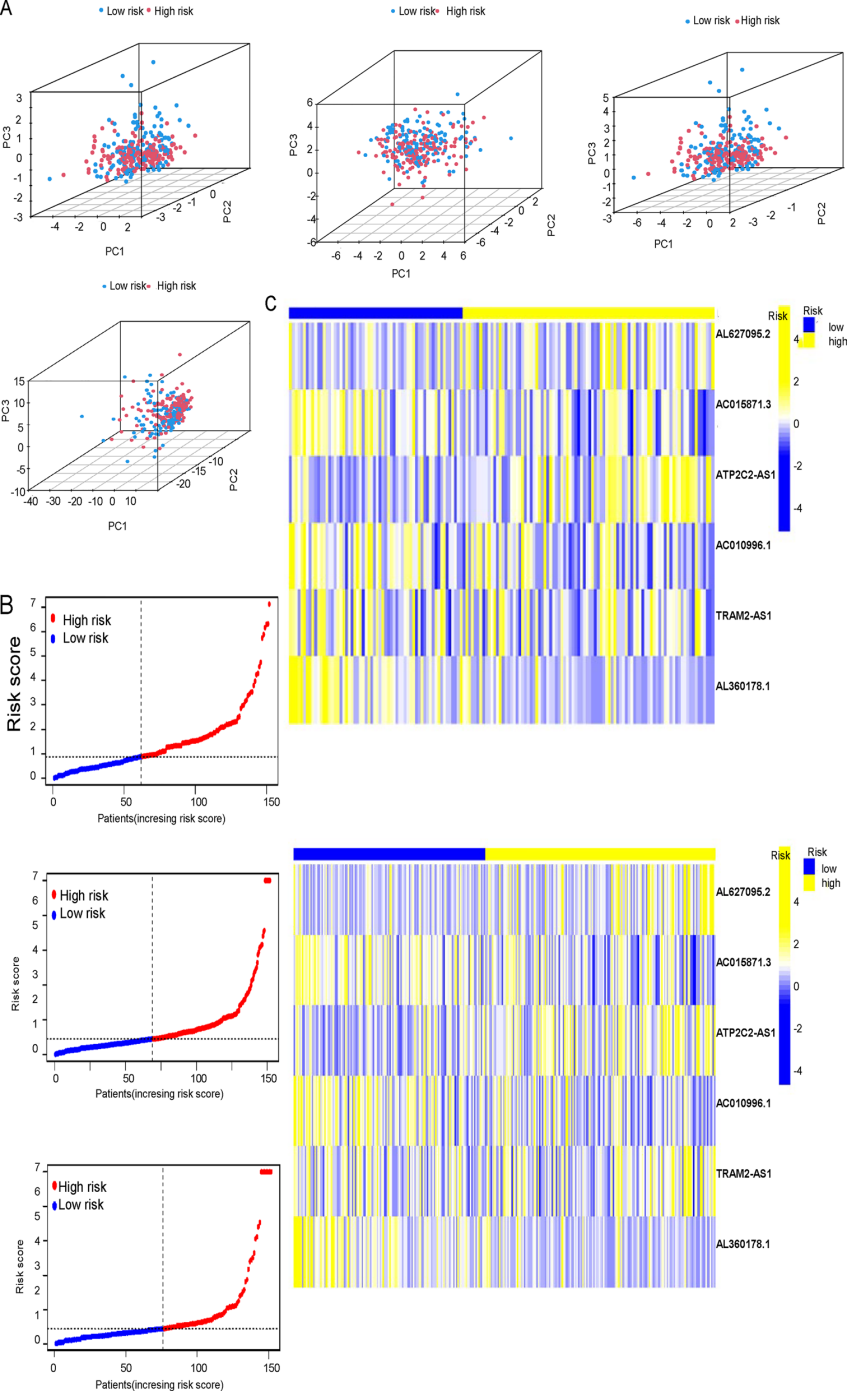
CUPIncRNA signature predicts prognosis in CESC. **A** Principal component analyses (PCA) of the expressions of all CRGs, all CUPIncRNA, OS-related CRGs, and 5 CUPIncRNAs associated with prognosis; Principal component analysis (PCA) plot showing the distribution of gene expression patterns among all CRGs, all CUPIncRNA, OS-related CRGs, and 5 CUPIncRNAs associated with prognosis. **B** Distribution of the risk scores for CESC patients, based on the expression levels of the 5 CUPIncRNAs associated with prognosis. **C** Heatmap showing the expression levels of the 5 CUPIncRNAs associated with prognosis in the test and train, survival groups. The color scale represents the level of gene expression, with yellow indicating high expression and blue indicating low expression

**Fig. 3:**
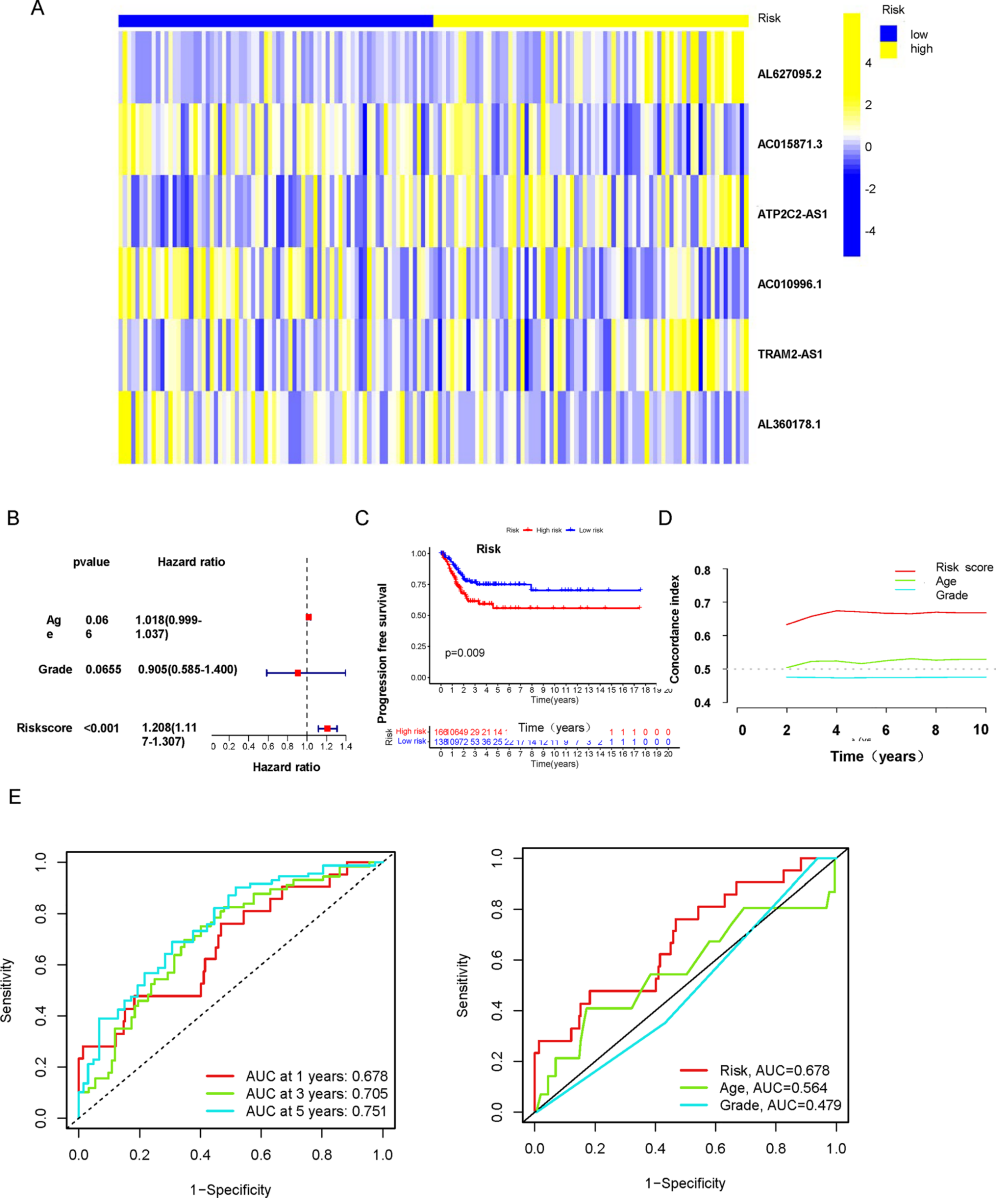
Prognostic Significance of CUPIncRNAs and Clinical Features in CESC. **A** Heatmap of five lncRNAs in overall group; Heatmap showing the expression levels of the 5 CUPIncRNAs associated with prognosis in the overall survival groups. The color scale represents the level of gene expression, with yellow indicating high expression and blue indicating low expression. **B** Multivariate Cox regression analysis of age, grade, and risk score showing their independent prognostic significance for overall survival in patients with CESC. **C** Kaplan–Meier curves showing the progression-free survival of patients with CESC, stratified by risk score. **D** C-index values for clinical features, including age, grade, and risk score, indicating their ability to predict overall survival in patients with CESC. **E** Receiver operating characteristic (ROC) curve for the prognosis model based on the 5 CUPIncRNAs associated with prognosis, demonstrating its predictive accuracy for overall survival in patients with CESC; Area under the curve (AUC) curve of clinical features; ROC curve for the clinical features, including age, grade, and risk score, showing their predictive accuracy for overall survival in patients with CESC

Principal component analysis (PCA) of the expressions of all genes, cuproptosis-linked lncRNA, cuproptosis-related gene and risk lncRNAs were carried out (Fig. 2A). The risk was increased as the patient’s risk score was increased in the test group, the training group, and the overall group. The high- and low-risk groups separated better in 5 cuproptosis-related lncRNAs linked with prognosis, indicating a good prediction value in the prognosis model (Figs. 2B, C and 3A). We used cox regression analysis to analyze the clinical features and found that only riskscore was significant (p < 0.01) (Fig. 3B). The low-risk group had better progression-free survival than the high-risk group (Fig. 3C). C-index results showed that risk score was better than other clinical indicators as a prognostic index (Fig. 3D). The AUC at 1 years, 3 years and 5 years was 0.678, 0.705 and 0.751, respectively, which indicated that this model has a good prediction effect. Meanwhile, we found that risk had the highest AUC of 0.678, which was higher than age and grade (Fig. 3E).

### Prognostic analysis of clinical features

**Fig. 4:**
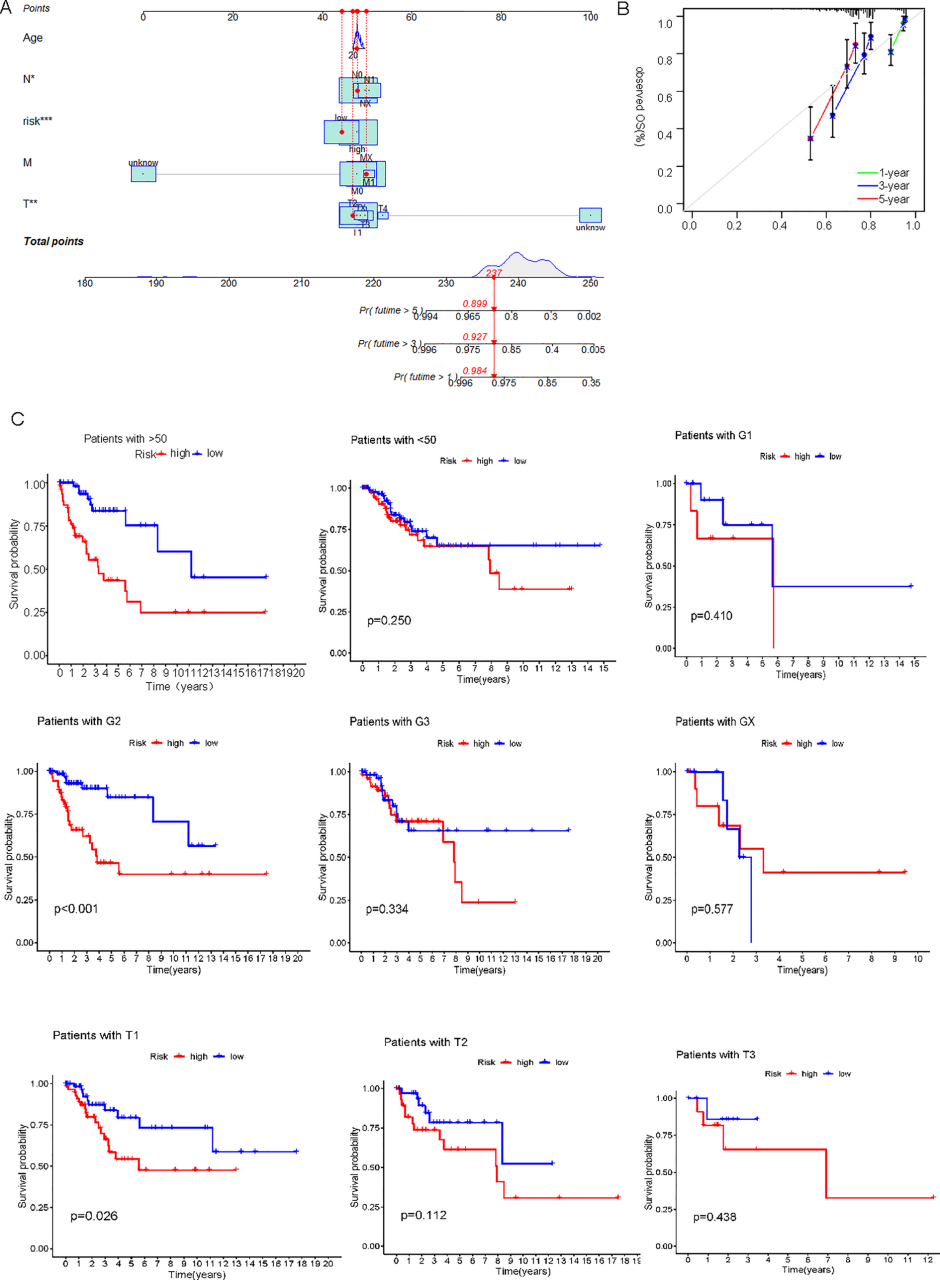
Nomograms and Survival Analysis for Predicting Overall Survival in CESC Patients. **A**, **B** Nomogram plots for predicting overall survival in patients with CESC, based on clinical features, including age, grade, and risk score. **C** Kaplan–Meier curves showing the overall survival of patients with CESC, stratified by clinical features, including age, grade, and tumor stage

**Fig. 5:**
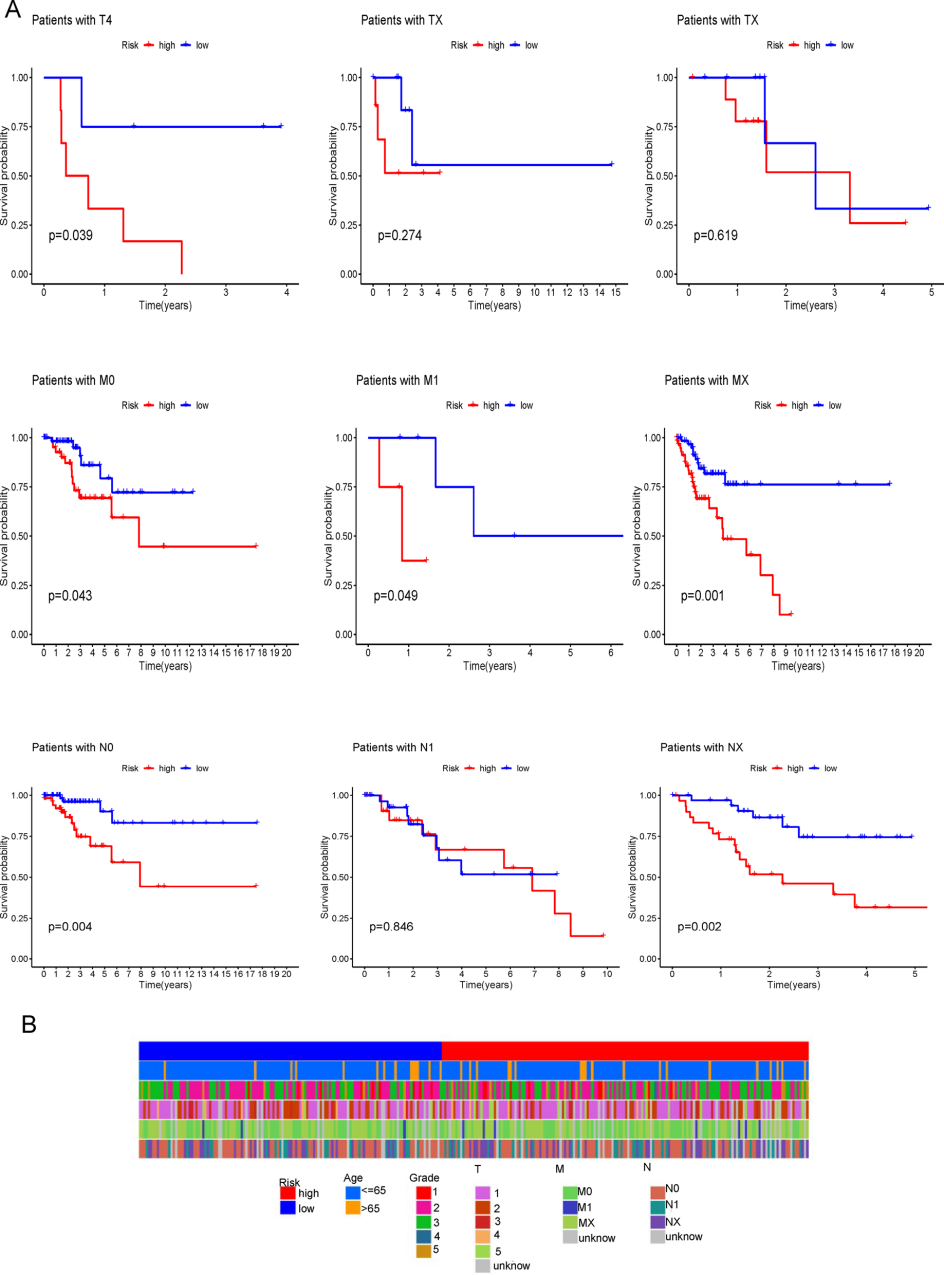
Clinical Feature Analysis in CESC: Survival and Expression; Kaplan–Meier survival analysis and heatmap of clinical features, including T, M, N, age, grade, and tumor stage, in patients with CESC. **A** Survival analysis of clinical features (T, M and N); Kaplan–Meier curves showing the overall survival of patients with CESC, stratified by clinical features. **B** Heatmap of clinical features; Heatmap showing the expression levels of clinical features, including age, grade, and tumor stage, in patients with CESC. The color scale represents the level of the feature, with red indicating high levels and blue indicating low levels

The results of nomogram and calibration curves of the nomogram were displayed (Fig. 4A, B). Prognostic analysis of the clinical features of cervical cancer was conducted by K-M analysis. The results showed that age, G, T, M and N could be used as good prognostic predictors (Fig. 4C). The low-risk group had a better prognosis than the high-risk group in the sample older than 50 years. Patients in G2, the high-risk group had a worse prognosis. Besides, there is a difference in the prognosis between high and low risk of T4. In MX, NO and NX patients, all low-risk groups had better prognosis than high risk groups (Figs. 4C and 5A). The clinical heat map was shown in Fig. 5B.

### Analysis of gene set enrichment

**Fig. 6:**
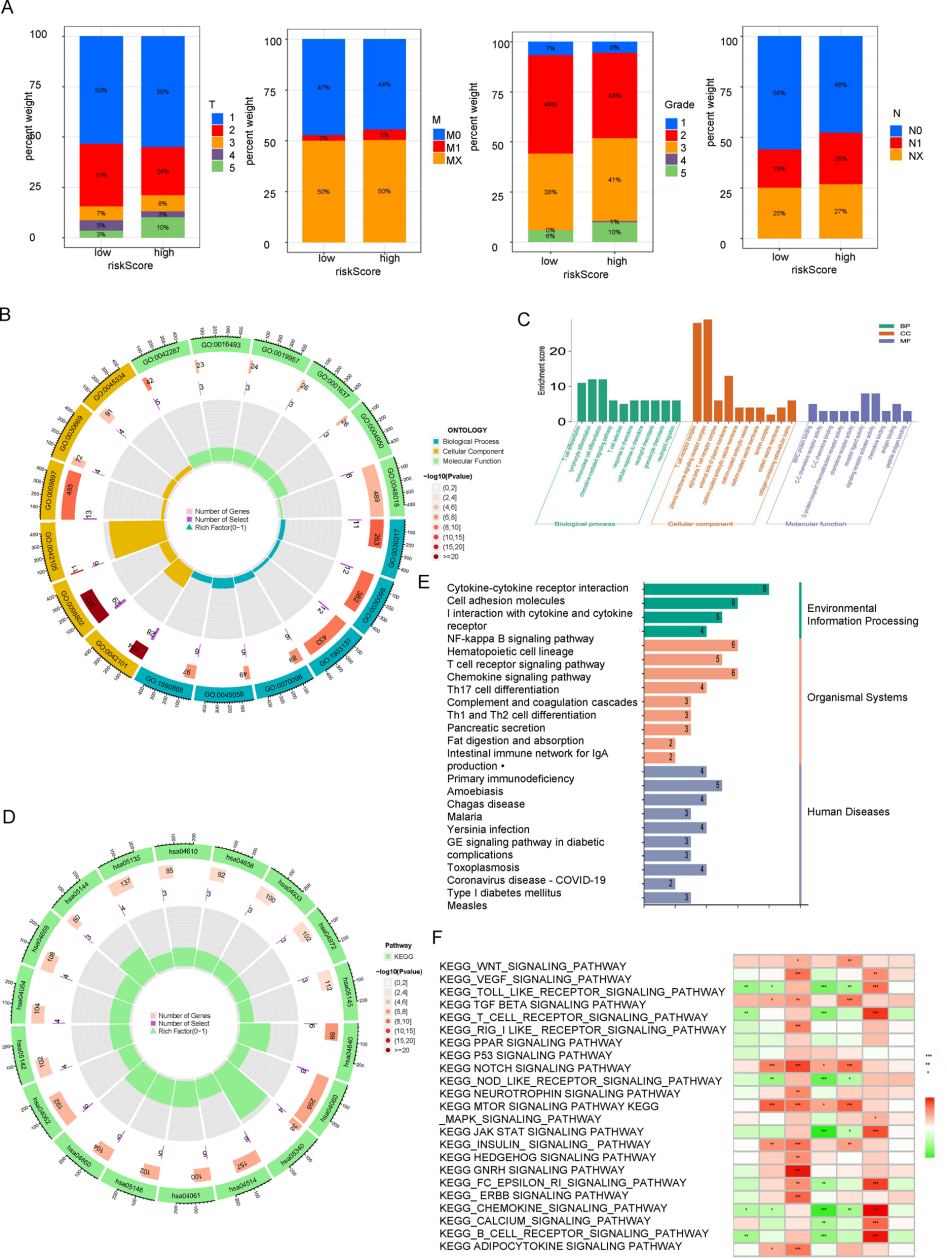
Analysis of Gene Expression and Pathway Enrichment. **A** Histogram showing the distribution of clinical features, including T, M, Grade and N, among patients with CESC. **B**, **C** Gene Ontology (GO) analysis showing the enriched biological processes, molecular functions, and cellular components associated with the differentially expressed genes in CESC. **D**, **E** Kyoto Encyclopedia of Genes and Genomes (KEGG) analysis showing the enriched pathways associated with the differentially expressed genes in CESC^32,33^. **F** Gene Set Variation Analysis (GSVA showing the enrichment of biological pathways and gene sets in patients with CESC based on their gene expression profiles

**Fig. 7:**
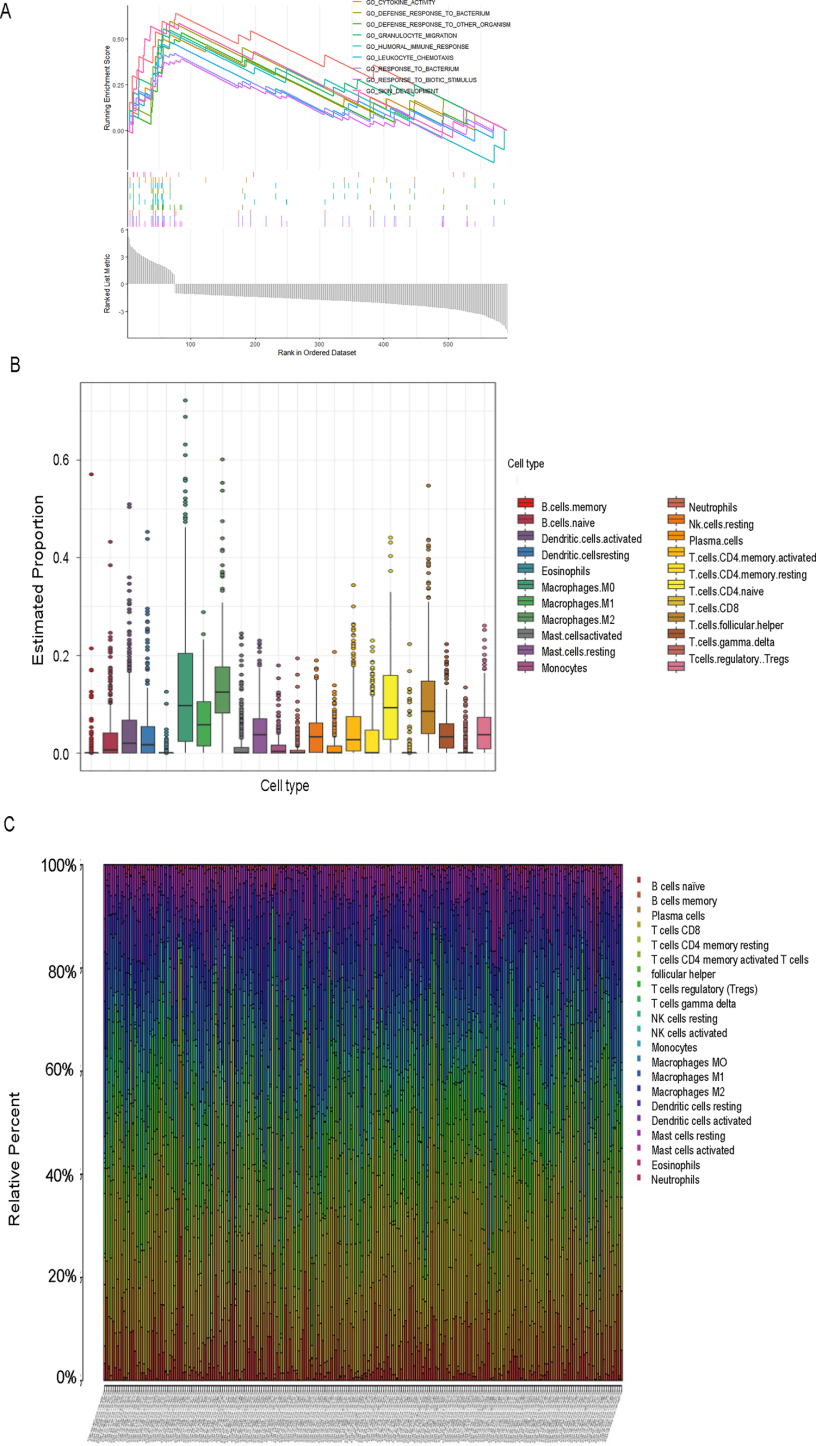
Analysis of immune infiltration and gene expression profiles in CESC. **A** Gene Set Enrichment Analysis (GSEA) analysis; GSEA analysis showing the enrichment of gene sets related to cuproptosis and immune-related pathways in patients with CESC. **B**, **C** Immune infiltration of 22 cells; Bar plots showing the relative abundance of 22 immune cells in patients with CESC, based on gene expression profiles, and their correlation with overall survival

The distribution of clinical characteristics in the high and low risk groups was also displayed (Fig. 6A). We used GO analysis for functional classification of genes or proteins into different biological functional domains. GO analysis has three main components: biological process (BP), cellular component (CC) and molecular function (MF). According to the results of BP in GO analysis, the genes were enriched in T cell differentiation, lymphocyte differentiation, mononuclear cell differentiation, chemokine-mediated signaling pathway, T cell selection, response to chemokine, cellular response to chemokine, neutrophil chemotaxis, granulocyte chemotaxis and neutrophil migration (Fig. 6B, C). Besides, in terms of CC, the finding of GO suggested that the genes were enriched in T cell receptor complex, plasma membrane signaling receptor complex, alpha–beta T cell receptor complex, external side of plasma membrane, clathrin-coated endocytic vesicle membrane, clathrin-coated endocytic vesicle clathrin-coated vesicle membrane, laminin complex, coated vesicle membrane, and collagen-containing extracellular matrix (Fig. 6B, C). MF primarily controlled MHC protein binding, C–C chemokine receptor activity, C–C chemokine binding, G protein-coupled chemoattractant receptor activity, chemokine receptor activity, receptor ligand activity, signaling receptor activator activity, chemokine binding, antigen binding, peptide antigen binding. There are three categories in KEGG analysis, including environmental information processing, organismal systems and human diseases (Fig. 6B, C). The results of KEGG analysis were displayed (Fig. 6D, E). Then, we used GSEA and GSVA to further explore the enrichment pathway. GSVA enabled the evaluation of underlying pathway activity variation in each sample, and it was used to explore the enrichment pathways of model-related lncRNAs, including AL627095.2, AC015871.3, STP2C2-AS1, AC010996.1, TRAM2-AS1 and AL360178.1. In total, 23 remarkably enrichment pathways were identified (Fig. 6F). The GESA results revealed that the following genes were enriched in the multiple pathways: GO _ CYTOKINE _ ACTIVITY, GO _ DEFENSE _ RESPONSE _ TO _ BACTERIUM, GO _ DEFENSE _ RESPONSE _ TO _OTHER _ ORGANISM, GO _ GRANULOCYTE _ MIGRATION, GO _ HUMORAL _ IMMUNE _ RESPONSE, GO _ LEUKOCYTE _ CHEMOTAXIS, GO _ RESPONSE _ TOBACTERIUM, GO _ RESPONSE _ TO _ BIOTIC _ STIMULUS, GO _ SKIN _ DEVELOMENT (Fig. 7A).

### Tumor microenvironment and immune infiltration

**Fig. 8:**
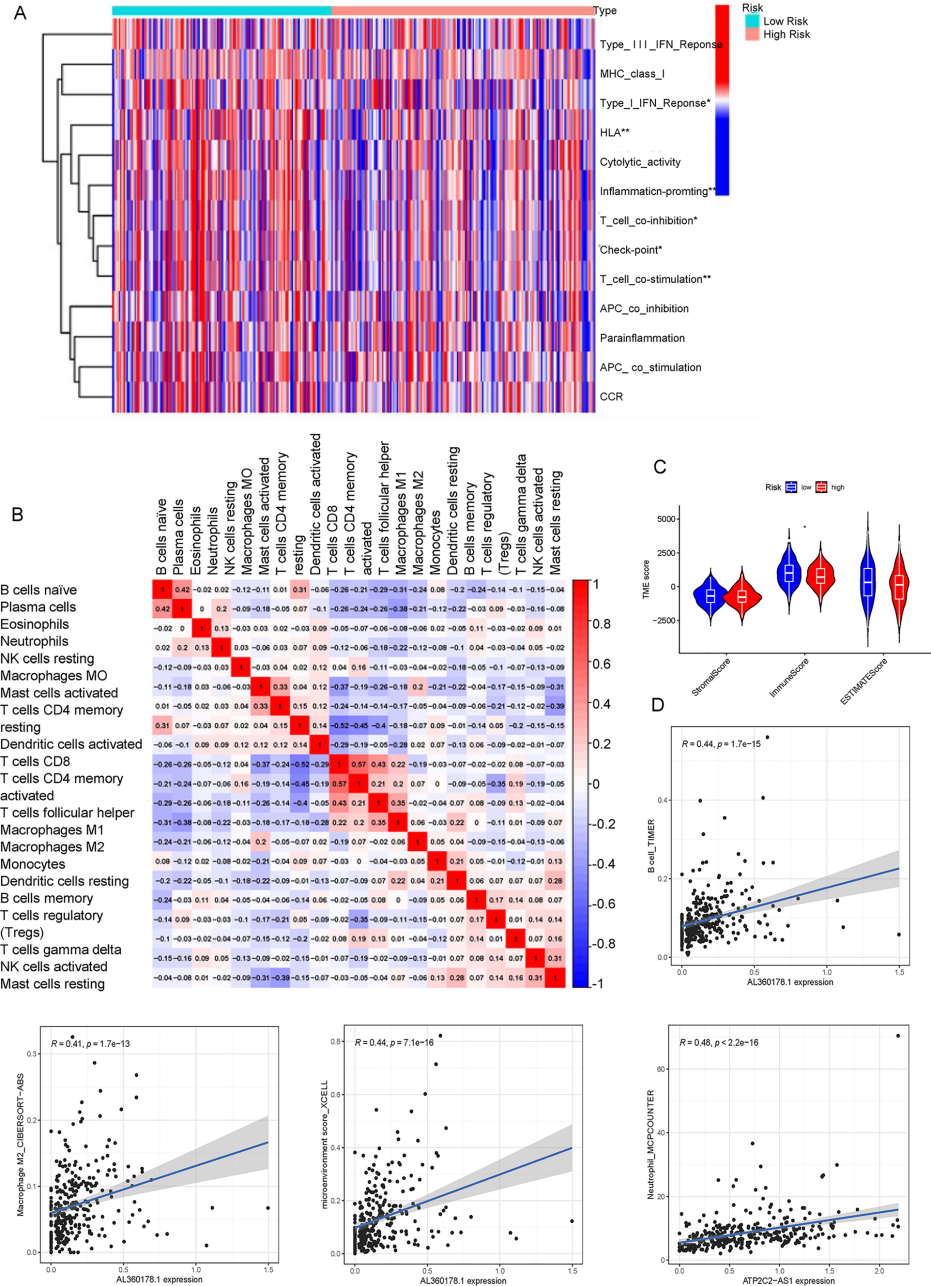
Gene expression analysis and immune cell profiling in CESC. **A** Heatmap showing the differential expression of immune-related functions between patients with high and low risk scores, based on gene expression profiles. The color scale represents the level of expression, with red indicating high levels and blue indicating low levels. Asterisks indicate statistical significance, with *p < 0.05, **p < 0.01, and ***p < 0.001. **B** Correlation matrix showing the relationship between the relative abundance of 22 immune cells in patients with CESC, based on gene expression profiles. The color scale represents the strength of the correlation, with red indicating positive correlation and blue indicating negative correlation. **C** Histogram showing the distribution of stromal score, immune score, and ESTIMATE score in patients with CESC, based on gene expression profiles. **D** Scatter plot showing the correlation between the expression levels of the five cuproptosis-related lncRNAs and the relative abundance of immune cells in patients with CESC, based on gene expression profiles. The x-axis represents the lncRNA expression level, and the y-axis represents the relative abundance of immune cells

**Fig. 9:**
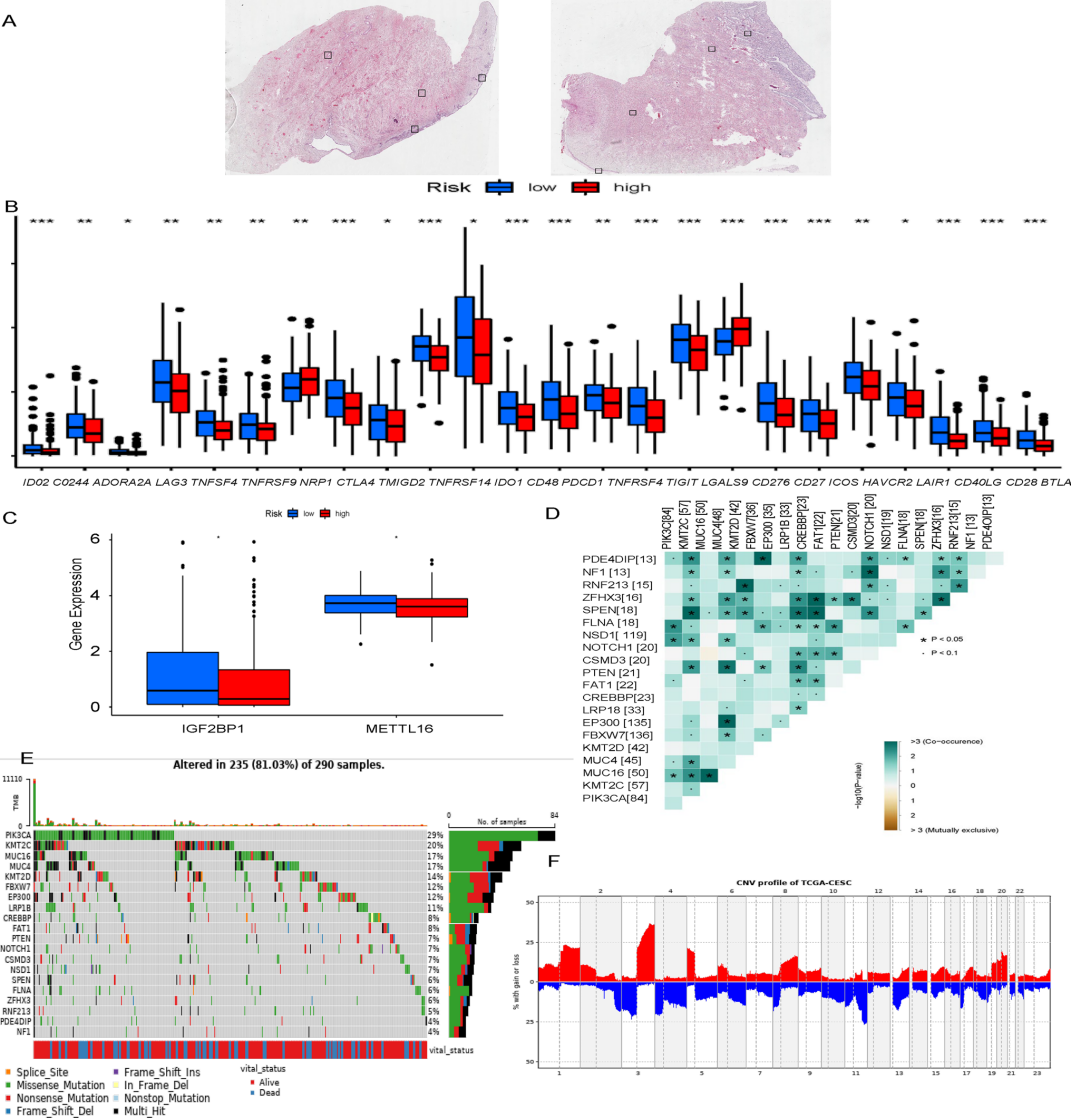
Molecular characteristics and biomarkers associated with cuproptosis and immune checkpoints in CESC. **A** Human Protein Atlas (HPA) analysis: Immunohistochemical staining images of cuproptosis-related lncRNAs and their corresponding proteins in cervical cancer tissues from the HPA database. **B** Comparison of the expression of immune checkpoints; Box plot showing the expression levels of immune checkpoints in patients with CESC, based on gene expression profiles. The y-axis represents the immune checkpoint gene expression level, and the x-axis represents the patient group. **C** Comparison of the expression of m6A-associated genes; Boxplot showing the differential expression of m6A-associated genes between CESC and normal cervical tissues. The significance levels are denoted by asterisks (*P < 0.05; **P < 0.01; ***P < 0.001) or "ns" for no significance. D Fisher exact test; Heatmap showing the enrichment of cuproptosis-related lncRNAs in the top mutated genes in patients with CESC, based on a Fisher exact test. The y-axis represents the -log10 P-value of the enrichment, and the x-axis represents the mutated gene set. **E** The tumor mutation burden; Heatmap showing the distribution of the tumor mutation burden in patients with CESC, based on whole exome sequencing data. **F** CNV profile; Heatmap showing the CNV profiles of cuproptosis-related lncRNAs in patients with CESC, based on gene expression profiles. The color scale represents the copy number status, with red indicating amplification, blue indicating deletion, and gray indicating no significant change

We used the estimate algorithm to calculate the immune infiltration of 22 types of immune cells in cervical cancer (Fig. 7B, C). Type 1 IFN response, inflammation promoting, T cell co-inhibition, check point and T cell co-stimulation were differentially expressed in the high-risk group and the low-risk group (Fig. 8A). Figure 8B presented the results of the association analysis of 22 different types of immune cells. The immune scores were higher in the low-risk group than in the high-risk group, but there was no difference in the stromalscore or estimate score (Fig. 8C). The expression of AL360178.1 was positively correlated with B cell and macrophage M2, with the correlation coefficients of 0.44 and 041, respectively. At the same time, the expression of AL360178.1 was positively correlated with the immune microenvironment score, and the correlation coefficient was 0.44, which indicated that the expression of AL360178.1 would affect the immune microenvironment of tumors (Fig. 8D). HPA results demonstrated the expression of PD-1 on cervical cancer tissues (Fig. 9A). Immune checkpoint results showed that a total of 26 immune checkpoints were correlated with gene expression (Fig. 9B). At the same time, the high- and low-risk groups were found to have differences in the expression of two m6A genes: IGF2BP1 and METTL16 (Fig. 9C).

### Analysis of tumor mutational burden

TMB is a quantitative biomarker reflecting the total number of mutations carried by tumor cells. The larger the TMB, the larger the mutation, the more easily the cancer cells can be detected by the immune cells and become the target of tumor immunity, which is likely to be effective for immunotherapy. The results of fisher exact test were displayed (Fig. 9D). 235 of the 290 samples were mutated, of which PIK3CA had the most mutation rate, accounting for 29%, and NF1 had the least mutation, accounting for 4% (Fig. 9E). Comprehensive analysis of copy number variation (CNV) was performed on cervical cancer patients, and the overview of CNV was presented (Fig. 9F).

### Analysis of drug prediction

**Fig. 10:**
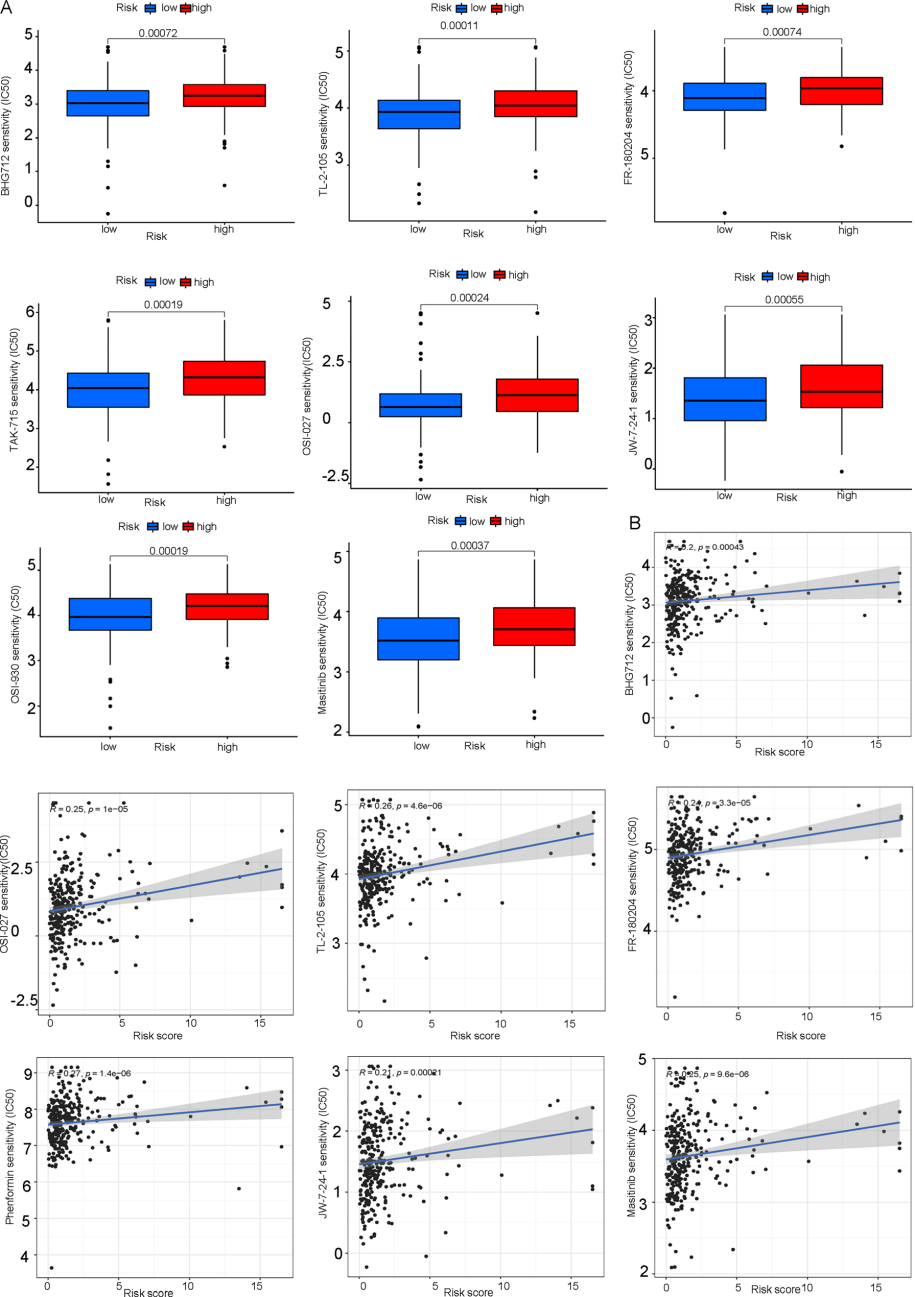
Association between drug sensitivity and riskscore in CESC patients. **A** Differences in IC50, estimated between the high- and low-risk groups are shown in the figure. IC50 represents the half-maximal inhibitory concentration of a drug. **B** The scatter plot displays the relationship between drug sensitivity and riskscore

**Fig. 11:**
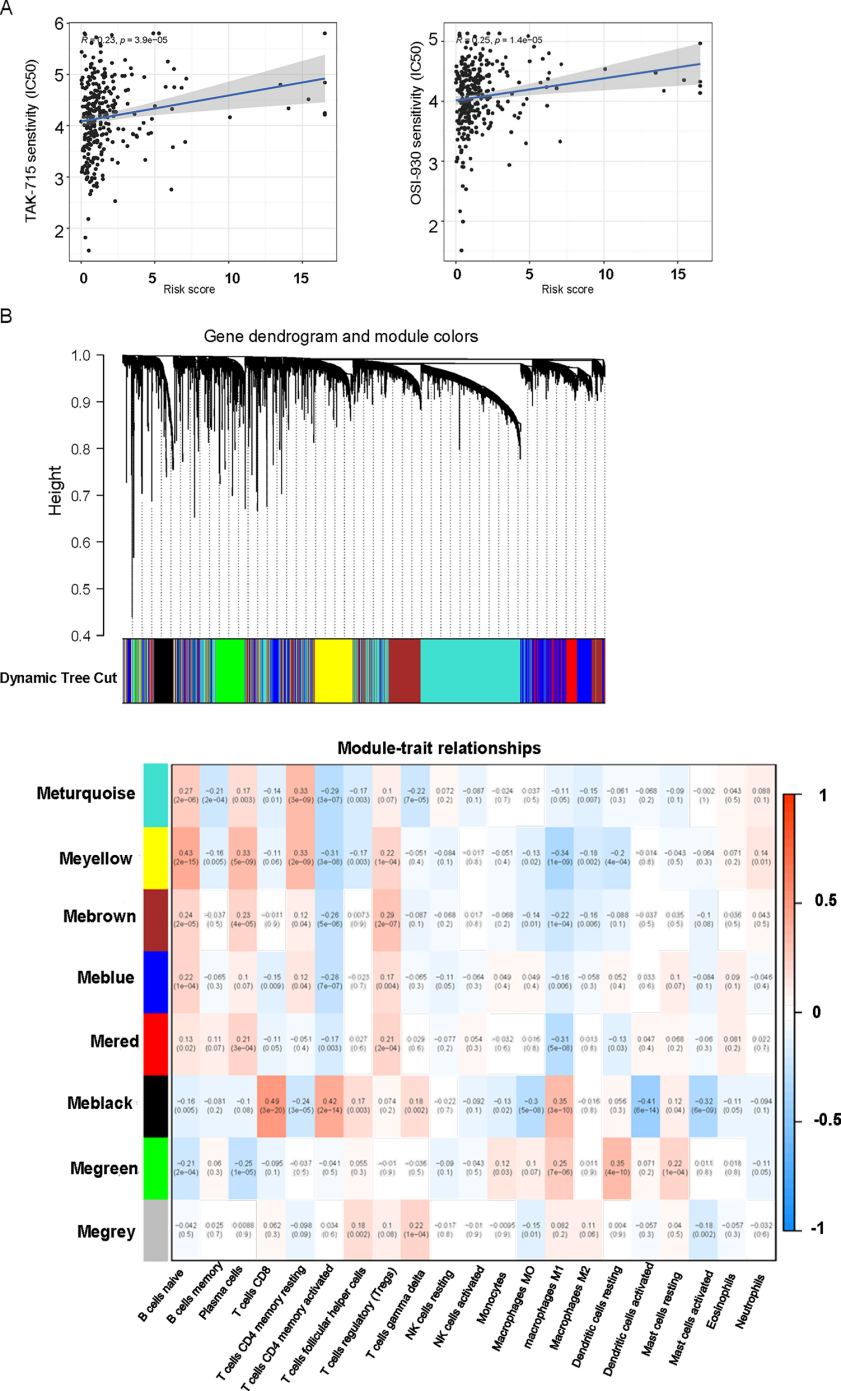
Analysis of gene co-expression and module detection using Weighted Gene Co-expression Network Analysis (WGCNA) to identify modules of genes related to drug sensitivity and their correlation with riskscore. **A** The scatter plot displays the relationship between drug sensitivity and riskscore. **B** WGCNA network and module detection: Selection of the soft-thresholding powers. The left panel showed the scale-free fit index versus soft-thresholding power. The right panel displayed the mean connectivity versus soft-thresholding power. Power 4 was chosen, for which the fit index curve flattens out upon reaching a high value (> 0.9). Cluster dendrogram and module assignment for modules from WGCNA. Genes were clustered based on a dissimilarity measure (1-TOM). Scatterplot of gene significance (y-axis) vs. module membership (x-axis) in the most significant module

We used the R package "pRRophetic" to evaluate prospective drugs according to IC50 values ​​of samples in the GDSC database so as to obtain more targeted therapy. The IC50 values of BHG712, TL-2-105, FR-180204, Masitinib, TAK-715, ODI-027, JW-7-24-2 and OSI-930 in the high-risk group were greater than those in the low-risk group, indicating that these drugs may be more appropriate for patients in the high-risk group (Fig. 10A). Furthermore, we found that these aforementioned drugs were positively correlated with riskscore (Figs. 10B and 11A).

### Analysis of WGCNA

In order to have relatively balanced scale independence and mean connectivity of the WGCNA, we undertook the investigation of network topology for various soft-thresholding powers. Power 4 was used to create a hierarchical clustering tree. Power 4 was the lowest power for which the scale-free topology fit index reached 0.90 (dendrogram). Then, 56 distinct gene modules were produced in the hierarchical clustering tree (dendrogram) via dynamic tree cut and merged dynamic, and each module was labelled by a different hue in the dendrogram. The module network dendrogram was created by grouping ME distances (Fig. 11B).

### Expression of LINC01833 and LINC02321

**Fig. 12:**
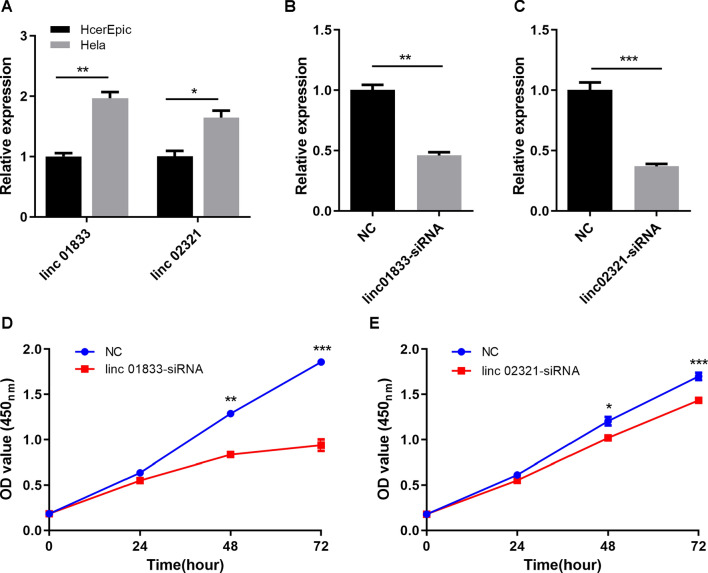
LINC01833 affects proliferation in HeLa cells. **A** Differential expression of LINC01833 and LINC02321 in HcerEpic and HeLa cells. **B**, **C** Validation of LINC01833-siRNA and LINC02321-siRNA expression levels in HeLa cells. **D**, **E** The impact of LINC01833 and LINC02321 knockdown on proliferation in HeLa cell

Firstly, we verified the mRNA expression of LINC01833 and LINC02321 in cervical cancer cell lines. We observed significant upregulation of LINC01833 and LINC02321 in cervical cancer cell lines indicating that LINC01833 and LINC02321 may be potential role in cervical cancer development (Fig. 12A).

### Validation of siRNA transfection efficiency

Compared to the negative control (NC), HeLa cells transfected with LINC01833-siRNA and LINC02321-siRNA showed a significant reduction in the expression levels of LINC01833 and LINC02321, indicating a pronounced knockdown effect (Fig. 12B, C).

### Proliferation and migration capability of cells after LINCRNA interference

**Fig. 13:**
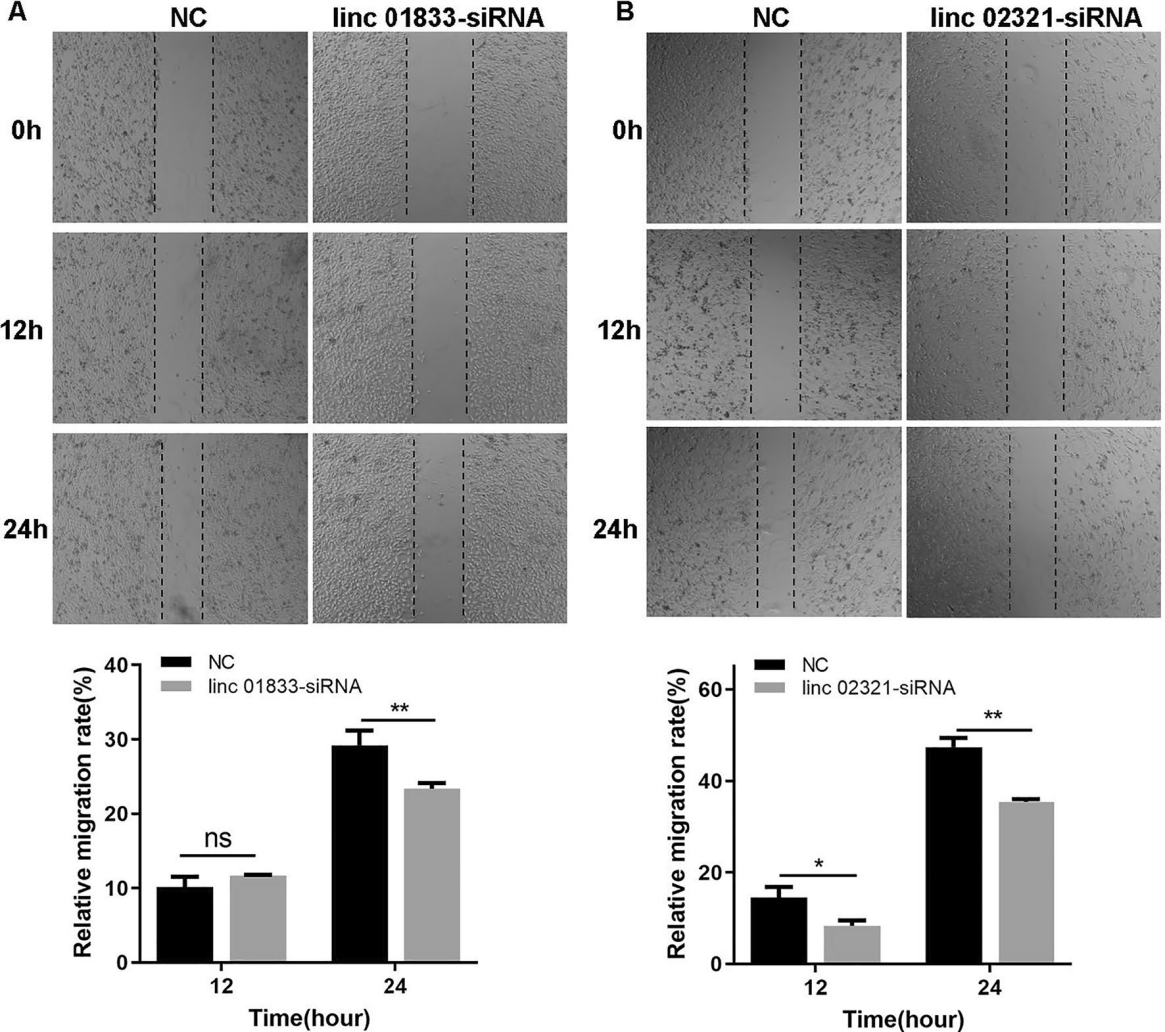
LINC01833 affects cell migration in HeLa cells. **A** The impact of LINC01833 knockdown on migration in HeLa cells. **B** The impact of LINC02321 knockdown on migration in HeLa cells

Compared to NC, the knockdown of LINC01833 and LINC02321 significantly reduced the proliferation capability of cervical cancer cells at 48 h and 72 h (Fig. 12D, E). Furthermore, compared to the NC, the knockdown of LINC01833 and LINC02321 significantly decreased the migration capability of cervical cancer cells (Fig. 13).

## Discussion

Cervical cancer is a type of cancer that affects the cervix, which is the lower part of the uterus that connects to the vagina [43]. Lymphatic metastasis is common at early stage, and the prognosis is relatively poor [44]. Therefore, it is vital to find new biological targets. We screened out 5 upregulated lncRNAs and 6 downregulated lncRNAs and identified cuproptosis-related lncRNAs, including LINC01833 and LINC02321. Afterwards, a prognostic model was constructed by multivariate COX regression analysis. Studies have shown that LINC01833 was associated with the prognosis of non-small cell lung cancer and bladder cancer [45, 46]. The screened LINC02321 had a considerably connection with the prognosis of bladder cancer, and markedly affected cell invasion and migration [47, 48]. However, the role of LINC01833 and LINC02321 was unelucidated in cervical cancer.

KEGG analysis revealed that selected lncRNAs were related to T cell receptor signaling pathway, Th17 cell differentiation, and Th1 and Th2 cell differentiation. Meanwhile, GO analysis also found that the selected lncRNAs were closely related to the biological process of T cells and neutrophil, including T cell differentiation, T cell selection, neutrophil migration. Therefore, we further evaluated the immune microenvironment in CESC. In the immune infiltration analysis, the level of immunoscore in the low-risk group was higher than that in the high-risk group. At the same time, the expression of AL360178.1 was found to be positively correlated with B cell, macrophages and tumor microenvironment score. Immune checkpoints are crucial for immune functions, and immune escape is the fundamental reason why tumour immunotherapy fails [49]. This work used immune checkpoint expression analysis to identify 24 immune checkpoint genes that were differentially expressed for cuproptosis-related lncRNAs between two risk groups. Our study has identified five genes associated with cuproptosis that are correlated with prognosis, providing crucial insights into predicting disease progression and guiding treatment strategies. Our findings are similar with previous research that has identified other prognostic genes, including AJ003147.1, CNNM3-DT, and SCAT2 [50–52]. These discoveries serve as a critical foundation and reference for further exploring the pathogenesis and pathophysiology of cuproptosis and for developing more effective treatments for patients. It is also essential for clinicians and scientists to consider the effects and impacts of these genes when assessing the prognosis and treatment options for cuproptosis.

As a class of protein modifications with more complex action modes and more diverse effects, ubiquitination plays an equally important role in all aspects of cell life cycle. Ubiquitin ligases play the important role in the specific recognition of target proteins and the regulation of the activity of the ubiquitination system [53–55]. An E3 ubiquitin ligase RING Finger 44 (RNF44) has been known to involve in carcinogenesis. RNF44 is a prognostic indicator of liver cancer, and its high expression is related to the poor prognosis of liver cancer [56]. Our data analysis found that RNF44 was correlated with AL360178.1, but the specific mechanism was unclear. Through literature searching, we found that there are few studies on the association between RNF44 and lncRNAs. Notably, one study suggested that lncRNA MIR600HG binds to miR-125a-5p, which targets RNF44. Down-regulation of MIR600HG increased the expression of miR-125a-5p and decreased the expression of RNF44 in oral squamous cell carcinoma (OSCC) [57]. LINC01833 is significantly upregulated in lung cancer, and LINC02321 is significantly upregulated in bladder cancer. Experimental studies have shown that LINC01833 and LINC02321 are also upregulated in cervical cancer, and interference with their expression through siRNA transfection significantly reduces cell proliferation and migration capabilities. This suggests that LINC01833 and LINC02321 could be potential risk factors associated with the development of cervical cancer. However, there are some limitations in this study. Only 19 cuproptosis-related genes were included in our study. The total number of cuproptosis-related genes is constantly changing, and there will be other cuproptosis genes in the future. The study only included the TCGA database, and larger datasets are needed. In vivo experiments are necessary to validate the role of cuproptosis-related genes. At the same time, the prognostic model needs to be validated with more clinical samples.

## Conclusion

In conclusion, based on the expression profile data of TCGA, we developed a model for predicting the prognosis of cervical cancer associated with cuproptosis. At the same time, this study explored the relationship between lncRNA, cuproptosis and immune microenvironment. This study provides new potential targets for the diagnosis and treatment of cervical cancer and brings new hope.

## Data Availability

All data, models, and code generated or used during the study are available from the corresponding author on reasonable request.

## Abbreviations

CCK-8: Cell Counting Kit-8
CESC: Cervical squamous cell carcinoma
circRNA: Circular RNA
CNNM3: Cyclin and CBS domain divalent metal cation transport mediator 3
CNV: Copy number variation
GO: Gene Ontology
GSVA: Gene set variation analysis
KEGG: Kyoto Encyclopedia of Genes and Genomes
lncRNA: Long noncoding RNA
ncRNA: Noncoding RNAs
OSCC: Oral squamous cell carcinoma
piRNA: Piwi-interacting RNA
PCA: Principal component analysis
ROC: Receiver operating characteristic
RNF44: RING Finger 44
SCAT2: S-phase cancer associated transcript 2
siRNA: Small interfering RNA
TMB: Tumor mutation burden
tRNA: Transfer RNA
TCA: Tricarboxylic acid
TME: Tumor microenvironment
WGCNA: Weighted correlation network analysis
